# Characteristics of Congenital Clasped Thumb: A Case Report and Literature Review

**DOI:** 10.3389/fped.2021.638059

**Published:** 2021-07-14

**Authors:** Sungmin Kim, Woo Kyoung Kwak, Sung Taek Jung

**Affiliations:** Department of Orthopedic Surgery, Chonnam National University Medical School and Hospital, Gwangju, South Korea

**Keywords:** congenital hand deformity, thumb deformity, thumb hypoplastic, congenital trigger thumb, tendon lengthening, conservative managements

## Abstract

Congenital clasped thumb is a progressive flexion and adduction deformity presenting with heterogeneous congenital abnormalities and syndromes. This deformity is usually accompanied by first web space narrowing and metacarpophalangeal joint (MPJ) laxity. Understanding the various features of the clasped thumb and making an accurate diagnosis is essential for treatment. Depending on the classification, treatment can vary from conservative to surgical. We describe the case of a bilateral clasped thumb with various characteristics, which were treated differently according to the disease type. The deformity of the clasped thumb was bilateral, and the patient had MPJ flexion deformity, flexor pollicis longus shortening, first web space narrowing, and MPJ instability, which were confirmed through a stress test. The left thumb was a complex type and was surgically treated, whereas, the right thumb was a flexible type, which was treated with splinting; the treatment showed promising results at 2 years post surgery. Diagnosis of the clasped thumb through thorough history taking, physical examination and considering its characteristics, and appropriate classification of the disease is essential for treatment. Furthermore, a stress test can identify MPJ instability in the congenital clasped thumb.

## Introduction

The congenital clasped thumb is a progressive flexion and adduction deformity presenting with heterogeneous congenital abnormalities and syndromes (1) and is due to a lack of extensor mechanism (2). The deformity is usually accompanied by features such as first web space narrowing and metacarpophalangeal joint (MPJ) laxity (3, 4). Understanding the various features and making an accurate diagnosis are essential for treatment. Treatment depends on the disease classification (3–5). Here, we report a case of a bilateral clasped thumb with various characteristics, which required different treatment strategies; the left thumb was surgically treated, and the right thumb was treated with splinting.

## Case Description

**Figure 1:**
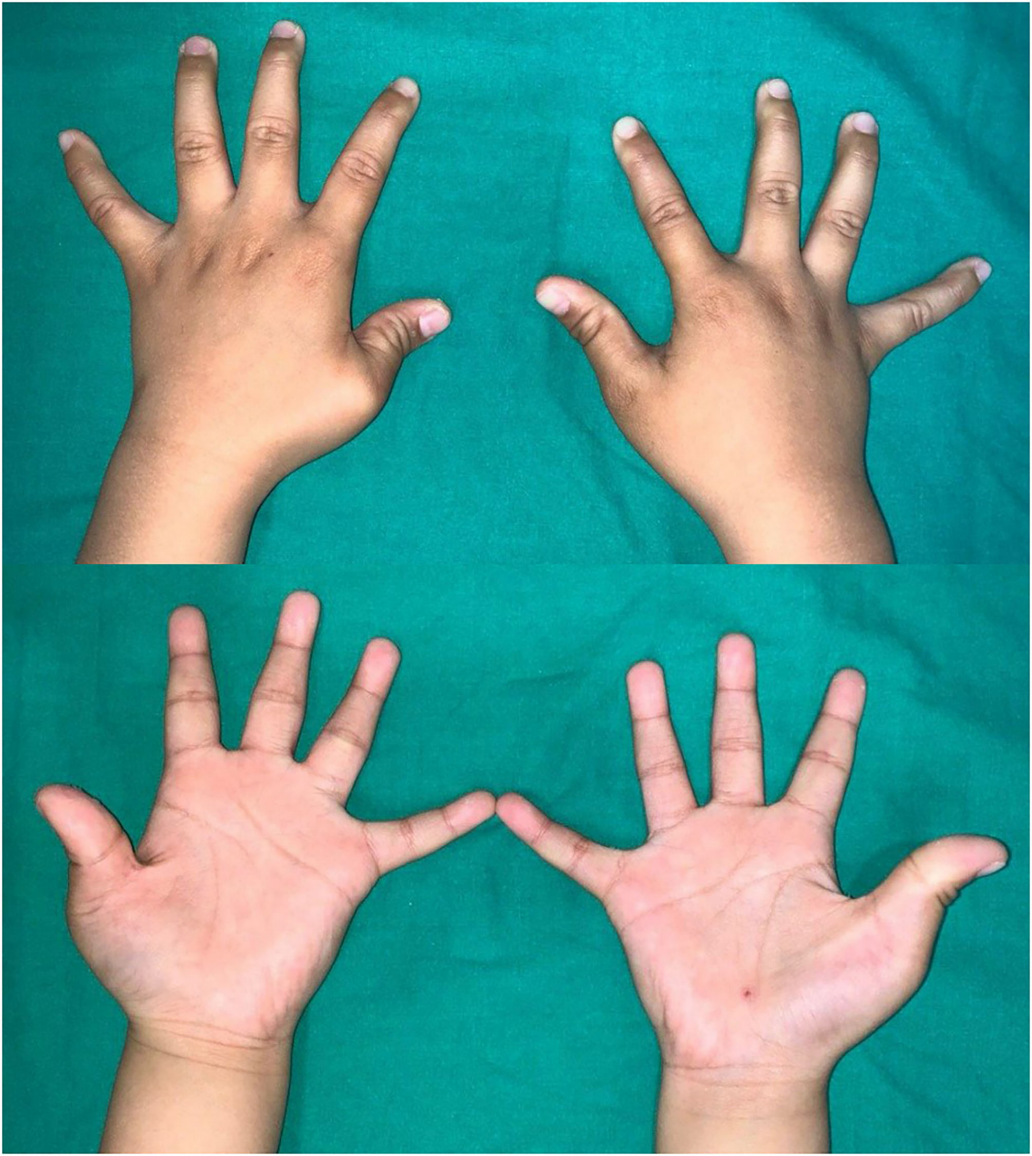
Adduction and flexion deformities on both thumbs.

**Figure 2:**
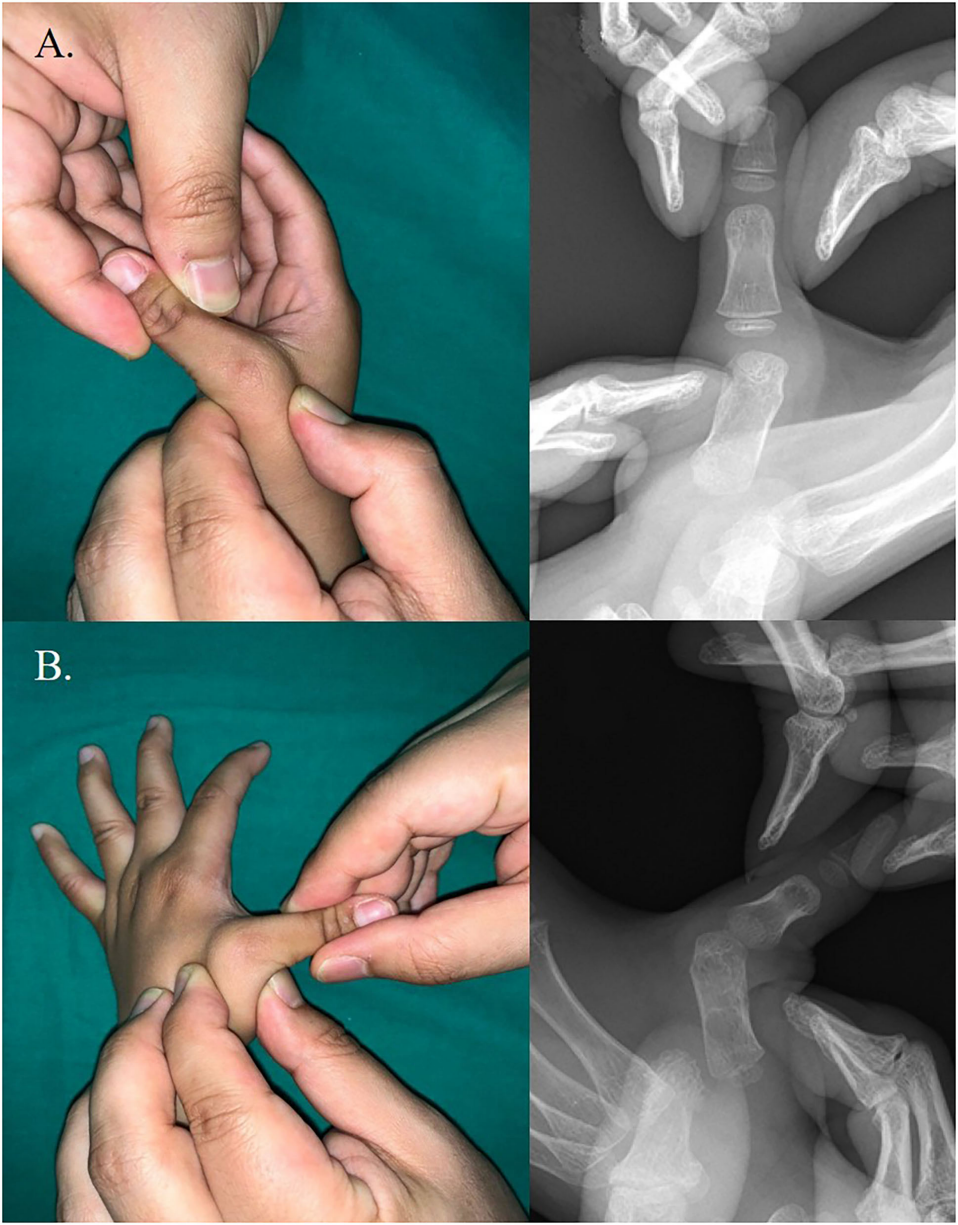
Metacaropophalangeal joint instability on stress test. **(A)** Right thumb. **(B)** Left thumb. A markedly unstable metacarpophalangeal joint was noted in left thumb.

In 2017, a 4-year-old boy presented to our institution with flexion deformities of his right and left thumbs. The child had undergone left trigger thumb release at 14 months of age at a local clinic. The parents noted that the left thumb deformity seemed to progress after surgery and that the right thumb also seemed deformed. The patient had difficulty in extending both thumbs from the palm. Physical examination revealed adduction and flexion deformity of both thumbs (Figure 1). The deformity was worse in the left thumb than in the right thumb. There was a transverse scar at the metacarpophalangeal crease of the left thumb. Flexion deformity of the MPJ and excessive hyperextension of the interphalangeal joint (IPJ) were noted. Extension of the MPJ was limited to 45° and radial abduction of the thumb was 10°. The IPJ deformity was passively correctible, but passive extension of the MPJ was limited due to contracture between the thumb and palm. Despite the IPJ deformity, active flexion and extension were noted, suggesting the presence and normal function of the flexor pollicis longus (FPL) and extensor pollicis longus (EPL) tendons. Passive extension of the MPJ combined with radial abduction of the thumb resulted in progressive flexion of the IPJ, signifying a shortening of the FPL tendon. While a mild laxity of the ulnar collateral ligament was noted in the right thumb, a marked MPJ instability was noted in the left thumb (**Figure 2**). Stress radiography to determine instability revealed that the left thumb showed >60° of instability compared with the right side (Figure 2). Extension of the MPJ of the right thumb was limited to 15°. However, the flexed MPJ and hyperextension of deformity at the IPJ were supple and passively correctible. The right thumb was flexible, but the left thumb had a fixed deformation; therefore, surgical treatment was advocated only for the left thumb. The right thumb was placed in a full-time splint extension for 6 months to achieve active extension.

## Surgical Procedure and Outcome

**Figure 3:**
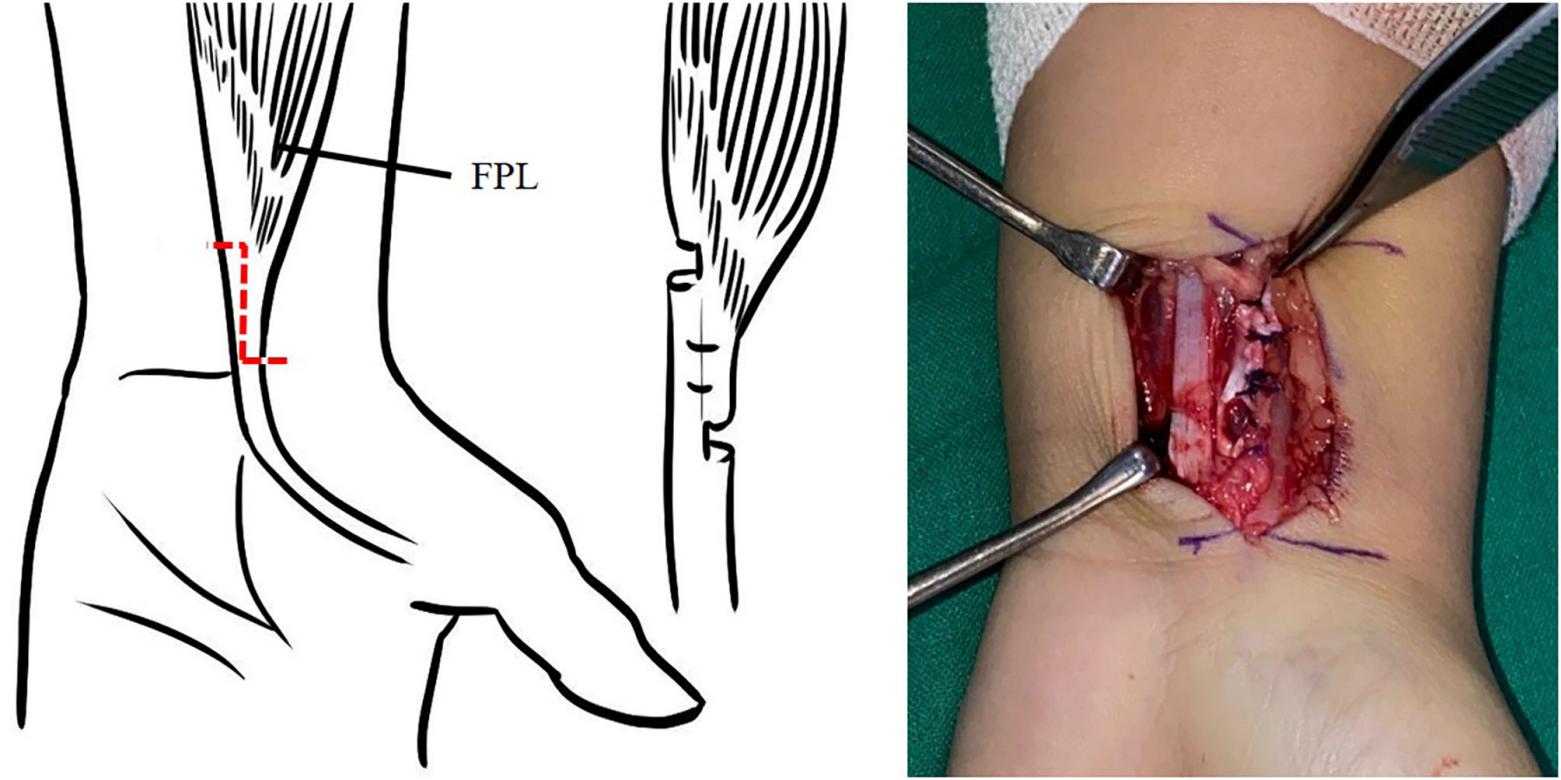
Flexor pollucis longus lengthening.

The surgical procedure for the left thumb addressed the following problems: (1) narrow first web space, (2) FPL shortening, (3) unstable MPJ, and (4) Extensor pollicis brevis (EPB) deficiency. Skin widening was achieved through four-flap Z-plasty. The dissection was deepened through the underlying fascia over the intrinsic muscles. After web space release, FPL lengthening was performed. A longitudinal incision was created over the FCR tendon proximal to the wrist crease to expose the musculotendinous junction of the FPL, and L-shaped lengthening was performed (Figure 3). With the thumb in radial abduction and the MPJ in full extension, the FPL tendon now rested against the bone with slight IPJ flexion. Subsequently, MPJ stabilization was performed using the breasting method of the capsule (3, 4) and ulnar collateral ligament. The MPJ was maintained in extension with a single k-wire. Tendon transfer was performed to restore active extension of the MPJ after its stabilization. The extensor indicis was used for transfer. The EIP was harvested through a longitudinal incision over the index MPJ and secured to the vestigial EPB tendon.

A long arm cast in a position with the thumb extended was applied immediately. The cast and k-wire were removed 6 weeks after surgery. The position was maintained in a long arm splint at night for another 4.5 months.

**Figure 4:**
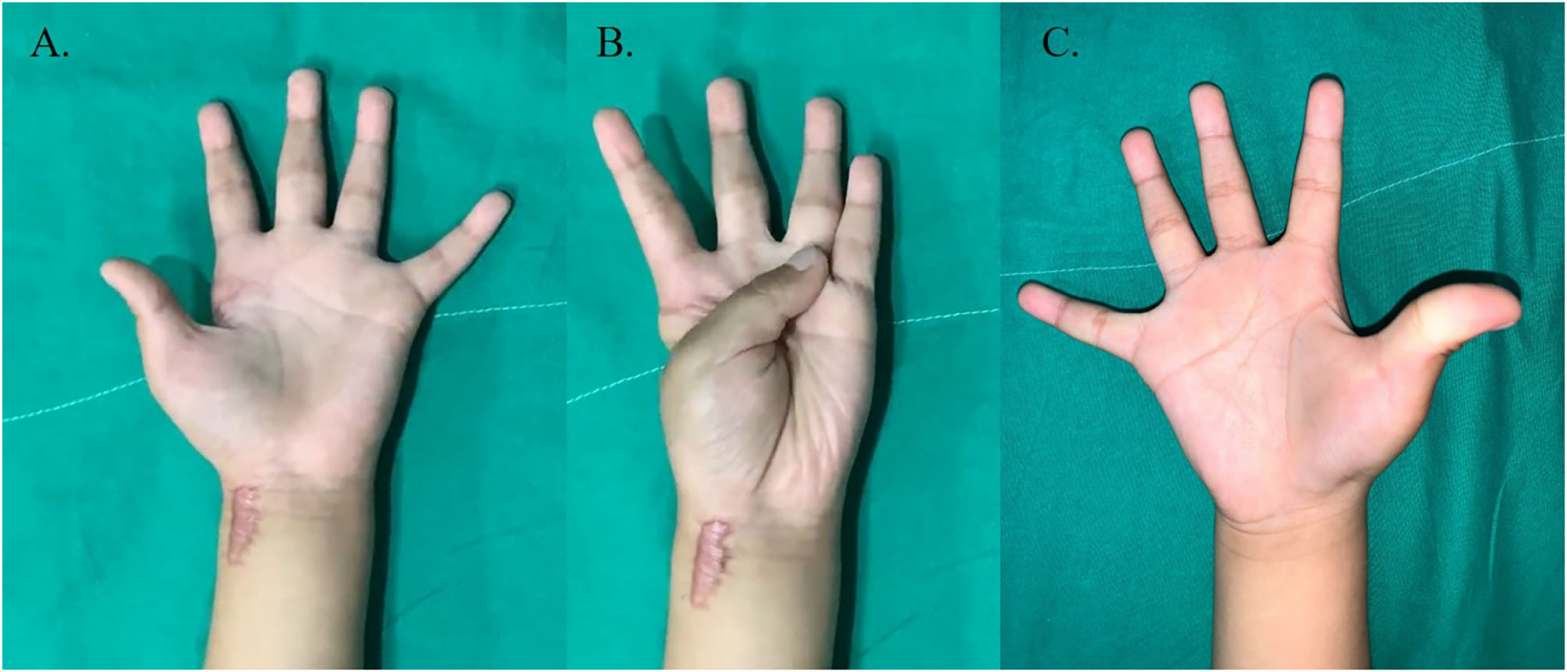
Clinical photographs of both thumb at 2 years post-treatment. **(A,B)** Left thumb showing opposition with ring finger. **(C)** Right thumb showing widened narrow web space.

Both thumbs were clinically evaluated 2 years post surgery. No limitation in range of motion was noted at the IPJ or MPJ of the thumbs, and the result was excellent, according to Weckesser et al. (6) staging. According to the Gilbert classification, abduction was 40–45°, and the rotation was 110–120° with stability (4). MPJ instability with stress test was within 10°. The thumb's opposition with other fingers was the ring finger with the left thumb and the little finger with the right thumb (Figure 4). The patient and parents were satisfied with the cosmetic appearance and function. Clinical data of the patient are shown in Supplementary Material 1.

## Discussion

Representative conditions that can cause flexion deformity of the pediatric thumb are congenital clasped thumb and trigger thumb. Trigger thumb and clasped thumb are characterized by flexion of the IPJ and MPJ, respectively (3, 4, 7–9). However, newborns hold the thumb in their palm until 3–4 months of age (10), and it is not easy to differentiate between these two conditions until the appropriate age to check the thumb's function is reached. The patient in this report was misdiagnosed with a trigger thumb, and after receiving the A1 pulley release, the flexion deformity gradually progressed. We attempted to evaluate the characteristics of the clasped thumb and review the literature (3–5, 7, 11–15).

Clasped thumb deformity at the MPJ is caused by an abnormal extensor mechanism (absence of EPB) and is typically bilateral (2). The flexed MCJ can be passively extended, but the patient cannot extend the thumb actively (9). If there is an EPL deficiency, flexed IPJ can be observed; however, it is passively extended. Therefore, when a clasped thumb is suspected clinically, it is important to evaluate the EPL's function. In our case, it was confirmed that the IPJ could be extended voluntarily with the MPJ extended. The trigger thumb has IPJ flexion that typically cannot be passively extended. There may be a palpable volar nodule (fusiform thickening of the flexor tendon) at the level of the MPJ in the trigger thumb (8, 14).

FPL shortening can be present in the clasped thumb. McCarroll (3) reported that the FPL tendon usually requires lengthening at the wrist level. Ghani et al. (4) reported that FPL shortening was observed in complex-type clasped thumb in a prospective study of 73 clasped thumbs. However, FPL lengthening was required in only two out of 20 thumbs that underwent surgery because MPJ chondrodesis was performed. Ruland et al. (14) reported the case of a patient with a clasped thumb who underwent FPL lengthening. The patient was misdiagnosed with a trigger thumb and underwent an A1 pulley release. They hypothesized that in the presence of an intact EPL, excessive release of the pulley system increased the moment arm of the FPL to create a boutonniere posture. Therefore, they performed A1 pulley reconstruction with FPL lengthening and reported good results. As in our case, the patient retained good function in the thumb without reconstruction of the A1 pulley system 2 years post surgery. Whether FPL lengthening is necessary in the clasped thumb depends on the operative findings.

A clasped thumb usually accompanies a narrow web space. Various procedures can be used to widen the first web space: Z-plasty or four-flap Z-plasty (16), butterfly flap (17), and modified dorsal rotational advancement flap (12). In our case, when the joint release was complete, four-flap Z-plasty was adequate. The choice of the technique is variable according to the degree of narrowing (3, 4).

MPJ instability is found in patients with clasped thumb (3, 4, 9, 13). MPJ instability is important not only because it differentiates trigger thumb but also because it should be addressed if extensor reconstruction is considered. McCarroll (3) reported that all their patients with fixed clasped thumb had gross laxity of the ulnar collateral ligament, which was fixed using capsular tissue. Lipskeir et al. (13) reported that MPJ stabilization was required in only 1 out of 19 patients with clasped thumb who underwent surgical treatment. Ghani et al. (4) reported that global instability of the MPJ was present in 18 hands and laxity of the ulnar collateral ligament of the MPJ was detected in two hands. In our case, we confirmed the MPJ instability through a stress test. Global instability was noted in the left thumb and mild laxity in the right thumb. We believe that stress test is helpful in detecting MPJ instability in patients with clasped thumb. Double-breasting procedure (3, 4) was performed, but not chondrodesis, in the left thumb; our patient remained remarkably stable and showed no recurrence signs 2 years post surgery.

Different treatment plan should be considered depending on the classification. Various treatment outcomes are shown in Supplementary Material 1. McCaroll et al. (3) classified clasped thumb into flexible and complex types. Flexible type can be treated by splinting, whereas surgery is necessary for complex type. Tsuyuguchi et al. (5) classified this deformity into three groups: group 1 (flexible type without contracture of soft tissue), group 2 (contracture of the palmar side of the thumb), and group 3 (relation with arthrogryposis). They reported good results in groups 1 and 2 cases with conservative methods. In groups 2 and 3 cases in which conservative treatment is ineffective, surgical treatment produces good results. Ghani et al. (4) reported no significant difference in severity and surgical results between groups 2 and 3 clasped thumbs; therefore, they were divided into supple and complex types. In our case, the right thumb was the flexible type and group 1. Excellent outcome was obtained with splinting for 6 months. However, as the left thumb was complex type and group 2, surgical treatment was required. We believe that familiarity with the assessment of disease severity and selection of appropriate treatment strategy are critical.

There is no standard timing for surgery for the collapsed joint of the thumb. Accurate diagnosis and disease stage is essential to determine whether surgical treatment is needed. In many cases, trigger thumb spontaneously resolves before the age of 1 (3, 8). Therefore, it may be helpful to have a sufficient period of observation until the child is 1 to 2 years of age or older and to evaluate extensor function. Tsuyuguchi et al. (5) performed surgery on clasped thumb in 10 hands, and the patient age range was 3–14. They reported that it is worthwhile to initially use conservative management since even patients with moderate deformities responded well to splinting within the 2 year age-group. They also reported that the neglected long-standing cases and cases with severe hypoplasia of the extensors should be treated by operative intervention in patients with group 2. Ghani et al. (4) reported the results of treatment in patients with clasped thumb and performed surgery in 28 hands and 17 patients, but did not specify the age of patients. They reported that conservative treatment was effective for patients under 1 year of age but then, and severe patients who do not respond to conservative treatment surgery gives better results.

## Conclusions

We report a patient with bilateral clasped thumb, which was treated with different treatment strategies. The left thumb had MPJ flexion deformity, FPL shortening, first web space narrowing, and MPJ instability, which were confirmed with a stress test. The left thumb was a complex type and surgically treated, and the right thumb was a flexible type and treated with splinting; both showed promising results at 2 years post surgery. Diagnosis of the clasped thumb through thorough history taking, physical examination, consideration of its characteristics, and proper classification of the disease is needed for treatment. Additionally, a stress test will help detect MPJ instability in congenital clasped thumbs.

## Data Availability Statement

The raw data supporting the conclusions of this article will be made available by the authors, without undue reservation.

## Ethics Statement

The studies involving human participants were reviewed and approved by Chonnam National Univeristy Hostpial Institutional Review Board. Written informed consent to participate in this study was provided by the participants' legal guardian/next of kin. Written informed consent was obtained from the individual(s), and minor(s)' legal guardian/next of kin, for the publication of any potentially identifiable images or data included in this article.

## Author Contributions

SJ contributed to manuscript conception, methodology, review, and supervision. SK examined the patient and contributed to manuscript conception, data curation, original draft preparation, and editing. WK contributed to data curation. All authors read and approved the final manuscript.

## Conflict of Interest

The authors declare that the research was conducted in the absence of any commercial or financial relationships that could be construed as a potential conflict of interest.
