# Supplementary material for: Characteristics of Congenital Clasped Thumb: A Case Report and Literature Review
Source: Front Pediatr. 2021 Jul 14;9:638059. doi: 10.3389/fped.2021.638059 (PMC8316600; doi:10.3389/fped.2021.638059)
Supplement: Supplementary file 1 [file Table_1.docx]

Table 1. Comparison of clinical data before and 2 years after surgery

|  | Before surgery | 2 years after surgery |
| --- | --- | --- |
| Limit of MPJ extension | Left: 45° | Left: <10° |
|  | Right: 20° | Right: <5° |
| Radial abduction | Left: 10° | Left: 40° |
|  | Right: 40° | Right: 40° |
| MPJ instability | Left: >60° | Left: <10° |
|  | Right: 10° | Right: <10° |
| Gilbert classification (4) | Left: Poor | Left: Excellent |
|  | Right: Fair to good | Right: Excellent |

MPJ: metacarpophalangeal joint
