# Supplementary material for: Characteristics of Congenital Clasped Thumb: A Case Report and Literature Review
Source: Front Pediatr. 2021 Jul 14;9:638059. doi: 10.3389/fped.2021.638059 (PMC8316600; doi:10.3389/fped.2021.638059)
Supplement: Supplementary file 2 [file Table_2.docx]

Table 2. Literature report on the treatment of clasped thumb

| Author | Year | Study design | Patients | Follow-up | Treatment |
| --- | --- | --- | --- | --- | --- |
| Tsuyuguchi et al. (5) | 1984 | Retrospective | 43  (75 hands) | 32 months | Operation on 10 patients (16 hands) |
| Lin et al. (14) | 1999 | Retrospective | 11  (17 thumbs) | N/A | Conservative management with splint |
| Ghani et al. (4) | 2007 | Prospective | 40  (73 hands) | 26 months | Operation on 17 patients (28 hands) |
| Ruland et al. (13) | 2012 | Case report | 6-year-old boy | 6 months | Operation |
| Sancar et al. (8) | 2015 | Case report | 4-year-old girl | 2 years | Operation |
| Smail et al. (11) | 2020 | Case report | 5-year-old girl | 17 years | Operation |

N/A: not applicable
